# 

**Published:** 1853-03

**Authors:** 


### CONTENTS

### OF NO III.

### ORIGINAL COMMUNICATIONS.

### ESSAYS AND CASES.

ART. I.—Discourse on the Life, Character, hnd Services, of Dr. Daniel Drake. Delivered, by request, before the Faculty and Medical Students of the University of Louisville, January 27, 1853, by S. D. GROSS, M. D., Professor of Surgery. 187

ART. II.—Acute Prostatitis, terminating in Suppuration, with complete Urethral and Perineal Fistulæ. By NAPOLEON B. ANDERSON, M. D., Louisville, Ky. 206

ART. III—A Case of Strangulated Hernia. By CHARLES FISHBACK, M.D., of Shelbyville, Ind. - - - 210

ART. IV.—Antiperiodic Properties of the Humulus Lupulus. By W. Y. GADBERRY, M. D., of Benton, Miss. - - 216

ART. V.—Resection of the Lower Jaw. Successfully Performed by Drs. GAINES & HENRY, of Hopkinsville, Ky. . 217

### REVIEWS.

ART. VI.—Principles of Chemistry. By B. SILLIMAN, JR., A.M., M.D., Professor of Chemistry and Toxicology in the University of Louisville. - - - - - - 221

ART. VII.—Transactions of the American Medical Association. 223

SELECTIONS FROM FOREIGN AND HOME JOURNALS.

Anæsthesia in Midwifery. By Henry Miller, M. D., Professor of Obstetric Medicine in the University of Louisville. - 235
The Speculum Uteri. By Henry Miller, M. D., Professor of Obstetric Medicine in the University of Louisville. - 241
An Account of the last Illness of the late Honorable Daniel Webster, Secretary of State: with a Description of the Post-mortem Appearances, &c. By John Jeffries, M. D. - 252
The Autobiography of Berzelius. - - - - 266
Substitute for Mercury in Syphilis. - - - 266
The Preservation of the Eyes, - - - - - 267

ORIGINAL INTELLIGENCE.

University of Louisville. - - - - - 271
American Medical Association. - - - - 272
Transactions of the Kentucky Medical Society. - - 272
Our Delay. - - - - - - - 275
Meteorological Phenomenon. - - - - 275
To the Medical Practitioners of Kentucky. - - 276
Certificates to Nostrums. - - - - - 276
Death of Eminent Physicians. - - - - - 277

